# Lactate in burn patients: biomarker of sepsis and mortality

**DOI:** 10.1186/cc10865

**Published:** 2012-03-20

**Authors:** A Mokline, L Gharsallah, A Abdenneji, H Oueslati, I Rahmani, B Gasri, I Jami, A Ghanem, A Messadi

**Affiliations:** 1 Burn and Trauma Center, Tunis, Tunisia

## Introduction

In this study, we attempted to assess whether the early plasma lactate (PL) level is a useful biomarker to predict septic complications and outcome in burn patients.

## Methods

A retrospective study was conducted in the burn care center in Tunis. Patients admitted within 24 hours from the thermal injury, from 1 January 2009 to 30 June 2010, were included. PL was measured early in the first 24 hours and controlled more than twice. For each measurement, 5 ml venous blood was drawn into a heparin-coated syringe. The normal lactate value was defined as 1 ± 0.5 mmol/l.

## Results

Over an 18-month period of study, 80 patients were enrolled. There were 60 males and 20 females. The mean age was 40.7 ± 19.5 and the average TBSA was 32 ± 21%. Upon admission, patients with an initial lactate value of more than 2 mmol/l were 86.7%. Fifty-eight percent of them have a lactate initial value of more than 4 mmol/l. In order to evaluate the potential impact of using early lactate measurements (H24 post burn injury) as predictor biomarker of sepsis in burn patients, a linear discrimination function was performed, by measuring the area under the ROC curve, and found that initial lactate value of more than 4 mmol/l provides the best sensitivity and specificity: 88% and 79% respectively. Also, the PL cut-off value for prediction of mortality was 4 mmol/l with a good sensitivity (86%) and specificity (92%). The area under the ROC curve was 0.96.

## Conclusion

Lactate appears to be a powerful predictor biomarker of sepsis and mortality in burn patients. A serum lactate of 4 mmol/l provides the best sensitivity and specificity.

